# Increased QPCT gene expression by the hepatitis B virus promotes HBV replication

**DOI:** 10.1371/journal.pone.0312773

**Published:** 2024-11-12

**Authors:** Conghui Zhang, Qingfeng Ma, Wei Wang, Hui Song, Xue Wang, Fengxia Xu, Chengliang Zhu, Xinghui Liu

**Affiliations:** 1 Department of Clinical Laboratory, Gongli Hospital of Shanghai Pudong New Area, Shanghai, China; 2 Dongming Community Health Service Center, Pudong New Area, Shanghai, China; 3 Department of Clinical Laboratory, Liyuan Hospital of Tongji Medical College, Huazhong University of Science and Technology, Wuhan, China; 4 Department of Clinical Laboratory, Wuhan Fourth Hospital, Wuhan, China; 5 Department of Clinical Laboratory, Renmin Hospital of Wuhan University, Wuhan, Hubei, China; Kwame Nkrumah University of Science and Technology, GHANA

## Abstract

Glutamine cyclase, an enzyme involved in posttranslational modifications, is encoded by the glutaminyl-peptide cyclotransferase (QPCT) gene. Gene microarray analysis revealed that the QPCT gene was highly expressed in HepG2.2.15 cells compared with that in HepG2 cells. The serum expression level of the QPCT gene was detected by ELISA and was significantly greater in HBV-infected patients than in healthy controls. The mRNA and protein expression levels of the QPCT gene were markedly greater in the HBV-expressing cell lines (HepG2.2.15, and HepG2 and Huh7 cells transfected with the pBlu-HBV plasmid) than in the HepG2 and Huh7 cells. The levels of HBV pgRNA and HBV-DNA copy number, as well as the levels of HBeAg and HBsAg, also increased in the HepG2 and Huh7 cell lines cotransfected with the QPCT gene expression plasmid and the HBV 1.3-fold plasmid. Our study indicated that HBV can promote the expression of the QPCT gene, which in turn promotes the expression and replication of HBV.

## Introduction

Chronic hepatitis B (CHB), caused by hepatitis B virus (HBV) infection, is a serious public health problem that has plagued humans for a long time, and no complete cure has been found yet [1]. HBV is an enveloped virus that contains an approximately 3.2 kb circular DNA genome, which includes four overlapping open reading frames (S, C, P, and X genes), and replicates genomic DNA by reverse transcription under viral reverse transcriptase action [2]. Chronic HBV infection leads to the progression of fibrosis, cirrhosis and liver cancer. The WHO estimates that 1.2 million people are infected with HBV every year, and 1.1 million people died of HBV infection in 2022 [3]. The current treatments for HBV infection fail to achieve a satisfactory reduction in morbidity and mortality in hepatocellular carcinoma [4]. This high mortality may be due in part to the fact that current treatments (*i*.*e*., nucleoside analogs) cannot wholly eradicate HBV infection [5]. Additionally, a long duration of administration is required for current treatments, worsening patients’ quality of life and imposing a heavy psychological burden. Therefore, accelerating research on HBV and clarifying its specific pathogenic mechanism is particularly imperative.

The QPCT gene encodes glutamine cyclase, which is involved in the post-translational modifications of proteins by converting the N-terminal glutamine of the protein to pyroglutamate. QPCT is resistant to protein degradation, highly hydrophobic, aggregation prone, and neurotoxic [6]. Glutamine cyclase is highly expressed in the brain and other peripheral tissues. Studies have confirmed that it is associated with numerous diseases, especially brain diseases such as Alzheimer’s disease, schizophrenia, and Huntington’s disease (HD) [7–9]; therefore, it may serve as an important new drug target. Studies have suggested that QPCT may be the QTL gene at chromosome 2p for the variation in spine bone mineral density (BMD) in the Chinese population [8], and QPCT gene variation is an essential factor for susceptibility to osteoporosis in adult females [9]. Studies have also revealed that there is a strong association between QPCT and certain cancers, such as melanoma [10], papillary thyroid cancer [11], renal cell carcinoma [12, 13], chronic lymphocytic leukemia [14], neuroblastoma [15], and cervical cancer [16]. Although the QPCT gene has been preliminarily studied in some diseases, there are no relevant findings in liver diseases. This study aimed to explore the association between abnormal expression of the QPCT gene and HBV infection and aimed to identify a new potential treatment for HBV infection.

## Materials and methods

### Plasmid construction

HBV-1.3 was generated in HepG2.2.15 cells and inserted into pBlue plasmids (Invitrogen) to construct the pBlue-HBV1.3 plasmid, which can produce infectious viral particles. The pcDNA3.1(+)3xFlag-QPCT plasmid expressing the full-length human QPCT was also constructed in our laboratory.

### Cell culture and transfection

The human hepatoma cell line HepG2 was obtained from the American Type Culture Collection (ATCC). The human hepatoma cell line Huh7 was purchased from the China Center for Type Culture Collection (CCTCC). The cells were cultured in DMEM supplemented with 10% fetal bovine serum (Gibco; Thermo Fisher Scientific, Inc.) and 1% penicillin‒streptomycin (Gibco; Thermo Fisher Scientific, Inc.) and incubated at 37°C with 5% CO_2_. The cells were transfected via Lipofectamine 2000 or Lipofectamine 3000 (InvivoGen) according to the manufacturer’s protocols.

### RT‒PCR and microarray analysis

Total cellular RNA was isolated via TRIzol reagent (CoWin Biosciences) according to the manufacturer’s instructions. Total RNA was reverse transcribed into cDNA via the use of oligonucleotides (DTs) as primers and glycerol-3-phosphate dehydrogenase (GAPDH) as a housekeeping reference gene. The following primers were used for RT‒PCR: QPCT.F/R: AGAATTACCACCAGCCAGCC/CCGGGTATCGCTCTATCAGC; APDH.F/R: GGGAAGCTCACTGGCATGG/TTACTCCTT GGAGGCCATGT; HBV pgRNA.F/R: TGGATTCGCACTCCTCCAGC TT/GGGACCTGCCTCGTCGTCTA. The difference in the results of the microarray analysis between the HepG2.2.15 and HepG2 cell lines was determined via the GeneChip™ Human Gene 2.0 ST Array (Applied Biosystems, USA). Furthermore, NetworkAnalyst (https://www.networkanalyst.ca/) was used to predict the genes associated with HBV replication.

### Enzyme-linked immunosorbent assay (ELISA)

This study was approved by the Ethics Committee of Pudong Gongli Hospital (No. 20200425–8), and participants signed informed consent forms. Eighty patients with HBV infection and 30 healthy individuals were enrolled in this study at Pudong Gongli Hospital (Shanghai, China) from June 1, 2020 to June 30, 2021. The ELISA kits for hepatitis B surface antigen (HBsAg) and e-antigen (HBeAg) detection were purchased from Shanghai Kehua Biological Engineering Co., Ltd., and the ELISA kit for QPCT detection was purchased from SAB (Signalway Antibody LLC). ELISA was performed according to the manufacturer’s protocols.

### Western blotting

Lysates from transfected HepG2 and Huh7 cells were prepared, denatured, and subjected to SDS‒PAGE. After electrophoresis, the proteins were transferred from the gel to a PVDF membrane. Five percent skim milk powder in TBST was used to block the PVDF membrane at room temperature for 1 h. Then, QPCT (Abcam, USA. ab201172) and GAPDH (Sigma, G9295) primary antibodies were added, and the samples were incubated overnight at 4°C. The corresponding HRP-labeled secondary antibodies (Sigma) were added and samples were incubated at room temperature for 1 h before images were acquired.

### Statistical analysis

GraphPad Prism 5 software was used for statistical analysis. Three separate experiments were conducted to verify the accuracy and reliability of the experiment. A t test was used for data analysis. A *P* value less than 0.05 was considered statistically significant. Significance was indicated as follows: * = *p* < 0.05; ** = *p* < 0.01; *** = *p* < 0.001; or ns (not significant) = *p*≥0.05.

## Results

### Bioinformatics analysis revealed that the QPCT gene is highly expressed in patients with HBV infection

**Fig 1:**
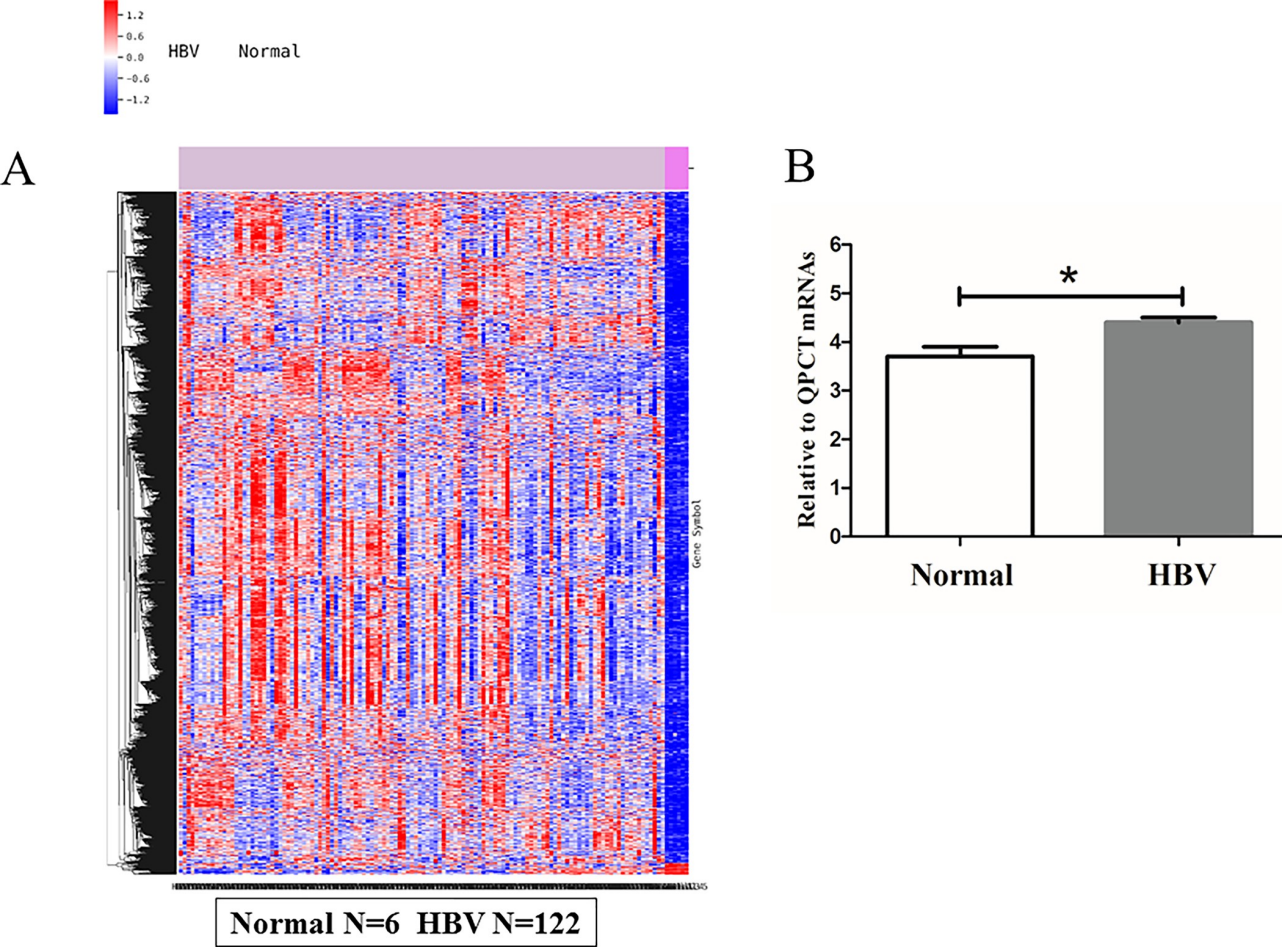
Bioinformatics found that the QPCT Gene was highly expressed in HBV patients. (A) The difference analysis of bioinformatics for QPCT expression in the GEO database between HBV patients and healthy controls. (B) QPCT mRNA expression difference between HBV patients and healthy controls.

**Fig 2:**
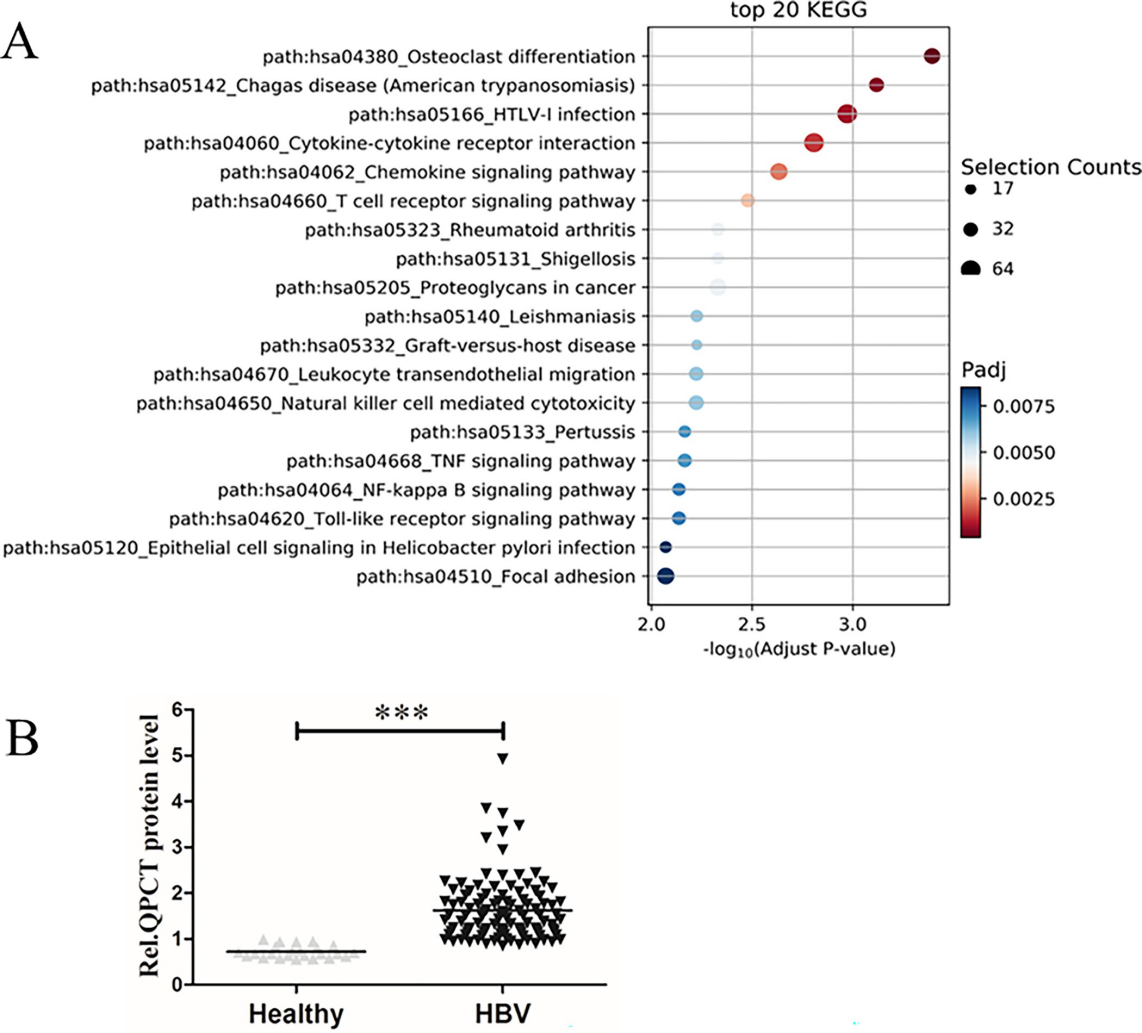
HBV infection promoted QPCT gene expression in hepatoma cells. (A) 20 downstream molecular pathways of QPCT. (B) HBV infection promoted QPCT expression in HBV-infected patients.

On the basis of the results of the gene chip, we conducted a bioinformatics analysis to detect the differences in QPCT expression in the GEO database between HBV patients and healthy controls (Fig 1A). We found that QPCT mRNA expression was significantly upregulated in HBV-infected patients compared with healthy controls (Fig 1B), which was consistent with the results of the gene chip. We also identified more than 20 downstream molecular pathways of QPCT (Fig 2A). This provides a good direction for further study of the molecular mechanism of QPCT in HBV pathogenesis.

### HBV promoted QPCT gene expression in HBV-infected patients

The results of the gene chip analysis indicated that the QPCT gene could be upregulated in HepG2.2.15 cells, and bioinformatics analysis revealed that the QPCT gene was highly expressed in HBV-infected patients. To further investigate the relationship between HBV infection and the serum level of QPCT, serum samples were collected from 24 healthy subjects and 83 HBV-infected patients, and the serum level of QPCT was determined with a commercial ELISA kit. The results showed that the expression of QPCT was significantly upregulated in HBV infected—infected patients compared with healthy controls (Fig 2B), which is consistent with the results of the gene chip analysis and our bioinformatics analysis.

### HBV infection promoted QPCT gene expression in hepatoma cells

**Fig 3:**
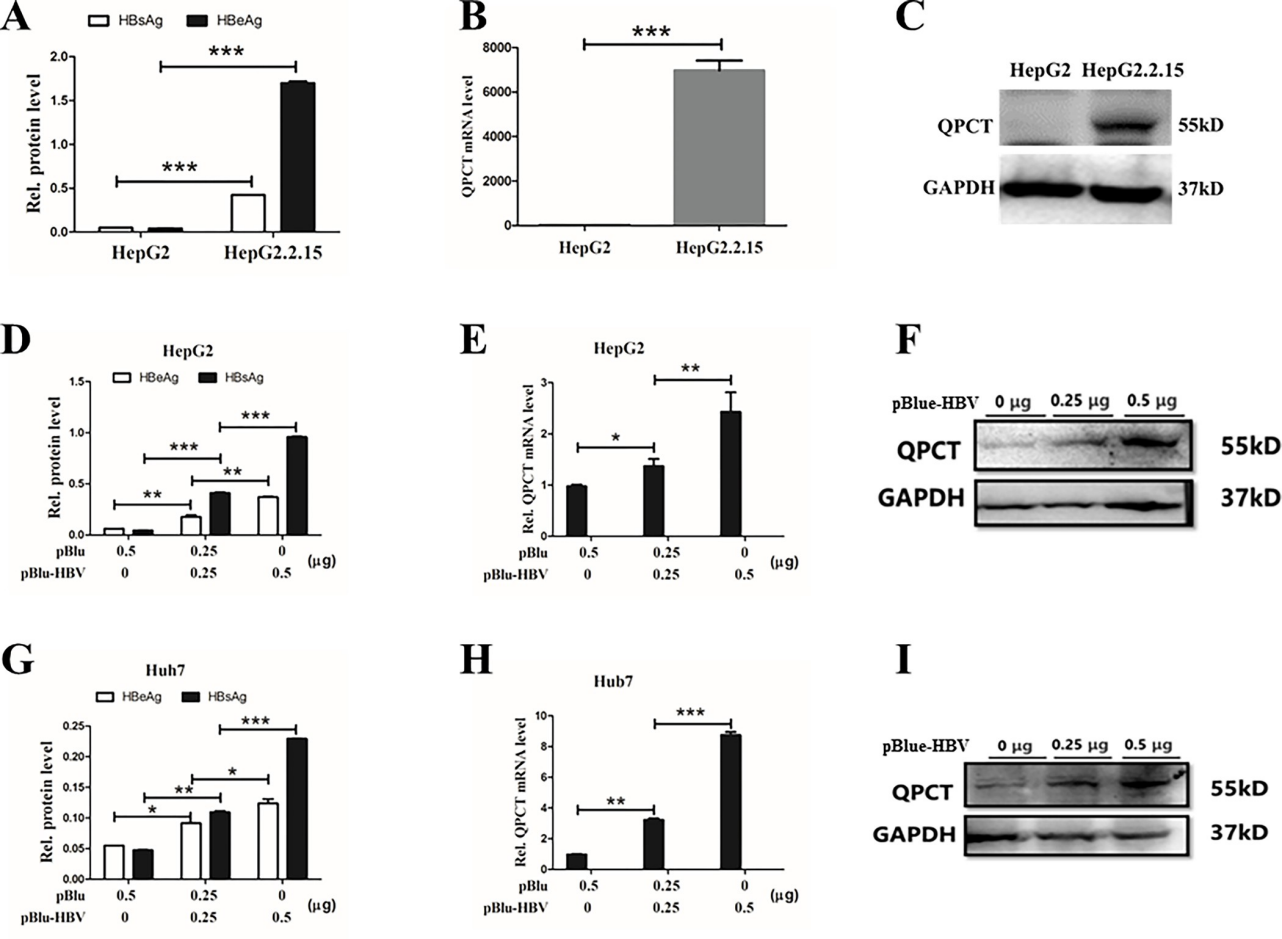
HBV infection promoted QPCT gene expression in hepatoma cells. (B and C) HepG2.2.15 cells and HepG2 cells at the same cellular concentration (1 × 10^6^ cells) were cultured for 48 h. Supernatants and cells were harvested. The protein and mRNA expression levels of QPCT were detected by Western blot and RT‒PCR, respectively. (E and F) After the gradient transfection of pBlue-HBV 1.3-fold plasmids into HepG2 cells, the cells were cultured for 48 h. Supernatants and cells were harvested. The protein and mRNA expression levels of QPCT were detected by Western blot and RT‒PCR, respectively, with the mRNA expression level of GAPDH as a reference. (H and I) Huh7 cells were analyzed in the same way as HepG2 cells. (A, D, and G) ELISA results for HBeAg and HBsAg concentrations in cell supernatants demonstrated successful infection.

**Fig 4:**
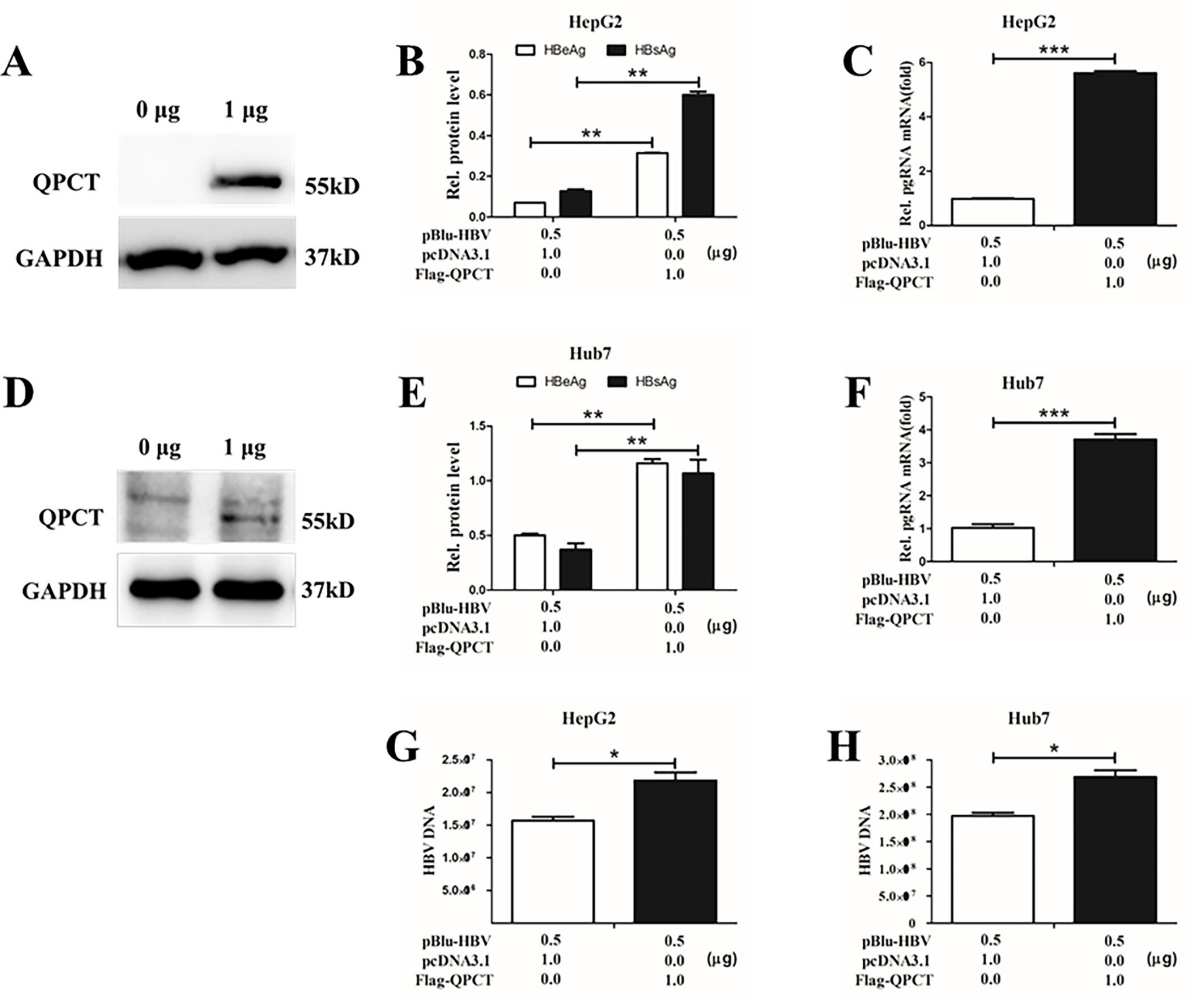
QPCT gene promoted HBV replication in hepatocytes. (A and D) HBV 1.3-fold plasmids and various concentrations of PcDNA3.1–3*Flag-QPCT were cotransfected into Huh7 and HepG2 cells and cultured for 48 h, supernatants and cells were harvested. (A and D) The overexpression of Flag-QPCT in the transfected HepG2 and Huh7 cells was detected by Western blot. (B and E) The expression levels of HBeAg and HBsAg in the supernatants were detected by ELISA. (C and F) Total RNA was extracted from the cells, and the mRNA expression of HBV pgRNA in the cells was detected by RT‒PCR, with the mRNA expression level of GAPDH as a reference. (G and H) Total DNA was extracted from the cells, and the HBV-DNA copies in Huh7 and HepG2 cells were detected by RT‒PCR.

To confirm whether HBV infection promotes QPCT gene expression, we performed pBlue-HBV1.3 plasmid gradient infection experiments in hepatoma cells. HBsAg and HBeAg levels in the culture medium of hepatoma cells were detected via ELISA, and the levels of QPCT mRNA and protein were detected via qRT‒PCR and Western blotting, respectively. We found that QPCT mRNA and protein levels in HepG2.2.15 cells stably expressing HBV (Fig 3A) were significantly greater than those in control HepG2 cells (Fig 3B and 3C). Additionally, HepG2 and Huh7 cells were subjected to gradient transfection with the pBlue-HBV1.3 plasmid, which can produce the HBV 1.3-fold genome in HBV-infected hepatoma cells. We found that the viruses replicated successfully in HBV-infected hepatoma cells. The expression levels of HBsAg and HBeAg in the culture medium were greater than those in the control plasmid pBlue group, and exhibited a gradient increase (Fig 3D and 3G). The mRNA (Fig 4E and 4H) and protein levels (Fig 3F and 3I) of QPCT also increased with increasing gradient of pBlue-HBV1.3 plasmids transfected into HepG2 and Huh7 cells. These results indicated that HBV infection could activate QPCT gene expression in hepatoma cells.

### QPCT promoted HBV replication in hepatoma cells

Although the QPCT gene plays an essential role in regulating various biological activities [17, 18], its function in HBV replication has not been reported thus far. We transfected the HBV 1.3-fold plasmid into Huh7 and HepG2 cells with or without the pcDNA3.1(+)3*Flag-QPCT plasmid (Fig 4A and 4D) and measured the expression levels of HBsAg and HBeAg in the supernatants via ELISA. The results revealed that the overexpression of the QPCT gene in Huh7 and HepG2 cells promoted the generation of HBV antigenic proteins (Fig 4B and 4E). The intracellular RNA of the transfected cells was isolated for reverse transcription, and the pgRNA level of HBV was detected via RT‒PCR after reverse transcription. The results revealed that the overexpression of QPCT in Huh7 and HepG2 cells promoted the expression of HBV pgRNA (Fig 4C and 4F). Moreover, the overexpression of QPCT also increased the number of HBV-DNA copies (Fig 4G and 4H). Overall, the overexpression of QPCT in HepG2 and Huh7 cells promoted the expression of HBV antigenic proteins and pgRNA and increased the number of HBV-DNA copies.

## Discussion

HBV is a noncytopathic human hepatotropic virus, and HBV infection can cause acute‒chronic hepatitis, severe liver failure and death [19], which is a global public health challenge with a scale comparable to that of tuberculosis, HIV and malaria [20, 21]. HBV infection is an independent risk factor for hepatocellular carcinoma (HCC), and CHB can progress to liver cirrhosis and further deteriorate into HCC. HCC accounts for 90% of primary liver cancer cases and is the third leading cause of cancer-related mortality worldwide [20–22].

Liver disease and liver cancer caused by HBV infection are still serious public health problems that researchers worldwide are committed to overcoming [23]. Encouragingly, some vaccines and antiviral drugs are available to alleviate the pressure of HBV infection on human health to a certain extent [24]. However, HBV RNA expression usually continues even during nucleoside analog therapy because free covalently closed circular DNA (cccDNA), a template for HBV RNA transcription, cannot be eliminated [25], which makes it more difficult to completely eradicate the virus. Therefore, basic science research is particularly important.

QPCT is an aminoacyl transferase of the transferase family that catalyzes chemical reactions and is associated with many human disorders [9]. In humans, the QPCT gene is located on chromosome 2P22.2 [22]. The QPCT protein is located in the Golgi complex, endoplasmic reticulum and secretory particles and plays an essential role in the maturation of different proteins [18]. It is a zinc-ion-dependent transferase with α-helix and β-sheet structures that is present in plants, animals and bacteria [24]. The gene microarray results revealed that QPCT gene expression was significantly higher in the HepG2.2.15 cell line than in the HepG2 cell line. Through bioinformatics analysis, we also found that QPCT was more highly expressed in HBV-infected patients compared with healthy controls, and some possible downstream molecular pathways of QPCT were also detected, which provided a direction for us to further study the molecular mechanism of QPCT with HBV. Moreover, the expression level of QPCT was significantly greater in serum samples from HBV-infected patients than in those from healthy subjects. As the experimental results at the cellular level confirmed, the QPCT gene was not only highly expressed in HepG2.2.15 cells but also significantly increased at the mRNA and protein levels with increasing HBV 1.3-fold plasmid concentration in Huh7 cells and HepG2 cells, indicating that HBV infection could promote the expression of the QPCT gene. It is still unclear whether increased expression of the QPCT gene may induce new biological effects, which is worthy of further exploration. Furthermore, when Huh7 and HepG2 cells were cotransfected with the HBV 1.3-fold plasmid and the QPCT expression plasmid, the pgRNA levels of HBV and HBV-DNA, as well as the levels of HBeAg and HBsAg, increased. Therefore, the upregulation of the QPCT gene could promote the expression and replication of HBV.

The mechanism of the chronic development of HBV infection is still unclear, but it is also an urgent problem that needs to be solved. Additionally, known as a "stealth" virus, HBV can trigger a negligible innate immune response early in infection, thereby promoting the establishment of chronic disease [26]. HBV infection can cause hepatitis B, which may progress to cirrhosis and liver cancer if not appropriately controlled. The cytokine CC ligand 2 (CCl2), a chemokine associated with tumor progression in a variety of cancer types [27–29], also known as human monocyte chemotactic protein 1 (MCP-1), is a member of the CC chemotactic family and a known chemokine that recruits monocytes/macrophages to sites of inflammation [30]. Chemokine activity is usually dependent on the modification of their nitrogen terminal. QPCT and its isoenzyme (ISOQC) have been reported to alter the N-terminus of CCl2. Although many studies have investigated the QPCT gene in tumors, relevant studies in liver cancer have not been reported. Uncontrolled and untreated CHB can progress to liver cirrhosis and even liver cancer. Therefore, we speculate that the upregulated expression of QPCT also enhances the modification of the N-terminus of CCl2 and leads to improved activity of CCl2 in CHB patients with cirrhosis and liver cancer, which promotes CHB progression and the development of liver cirrhosis and liver cancer. However, a limitation of this study is that, due to limited time and conditions, this experiment was not included. In future studies, we will further investigate the possible molecular mechanism from this perspective.

There are several other shortcomings in our study. Although we investigated the relationship between QPCT and HBV, the mechanism of QPCT in liver cirrhosis and hepatocellular carcinoma caused by HBV infection has not been explored in depth. Moreover, we discovered only some phenomena at the clinical and cellular levels, but an in-depth study of the molecular mechanism related to the expression of the QPCT gene promoted by HBV needs to be conducted. We will continue further investigations in subsequent analyses. We hope to increase efforts in the fight against HBV infection and identify promising treatments for HBV-infected patients in the future.

## Supporting information

S1 File (XLS)

S2 File (PPTX)
