# Supplementary material for: Increased QPCT gene expression by the hepatitis B virus promotes HBV replication
Source: PLoS One. 2024 Nov 12;19(11):e0312773. doi: 10.1371/journal.pone.0312773 (PMC11556691; doi:10.1371/journal.pone.0312773)
Supplement: S2 File — (PPTX) [file pone.0312773.s002.pptx]

## Slide 1
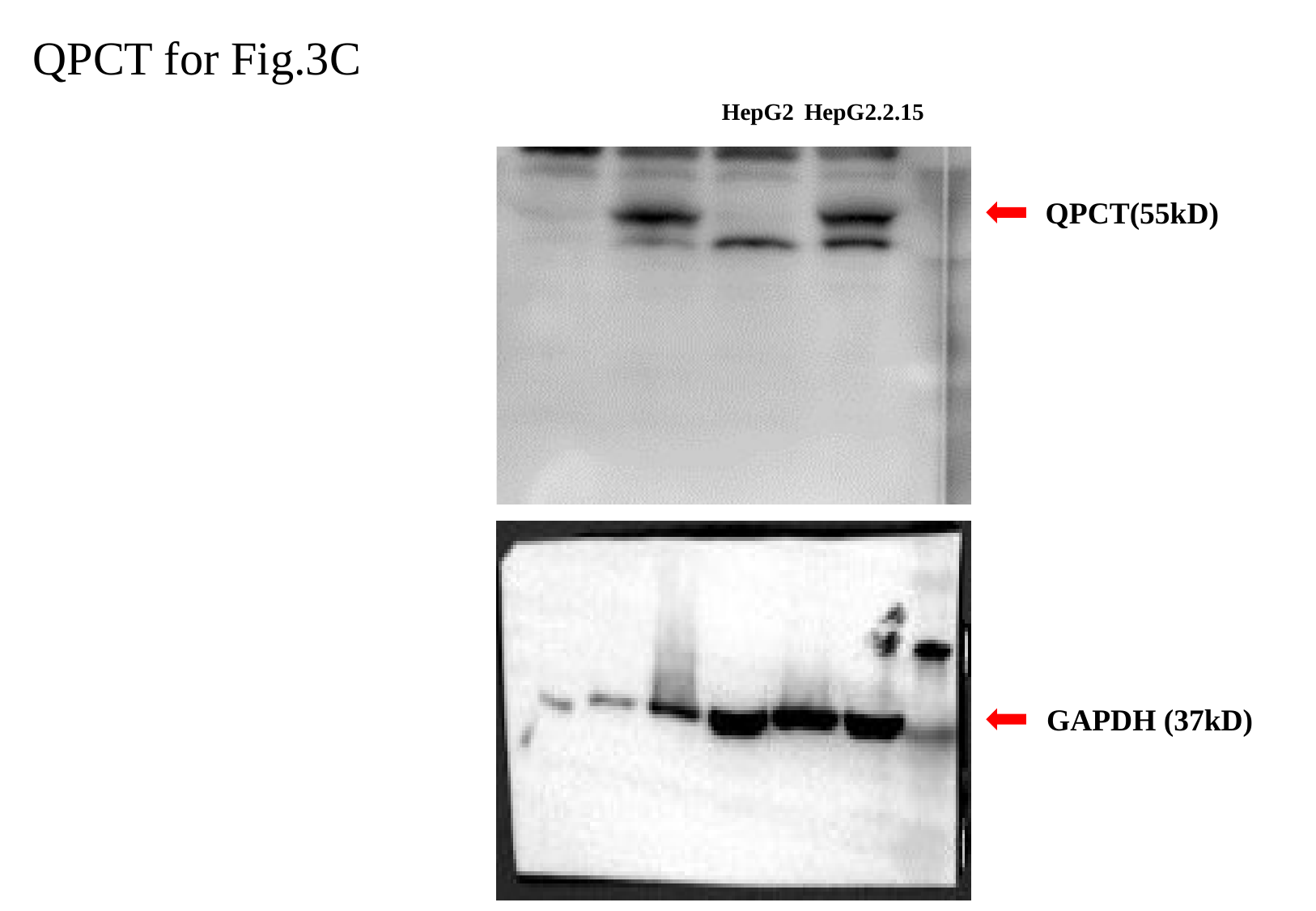

QPCT for Fig.3C
HepG2
HepG2.2.15
QPCT(55kD)
GAPDH (37kD)

## Slide 2
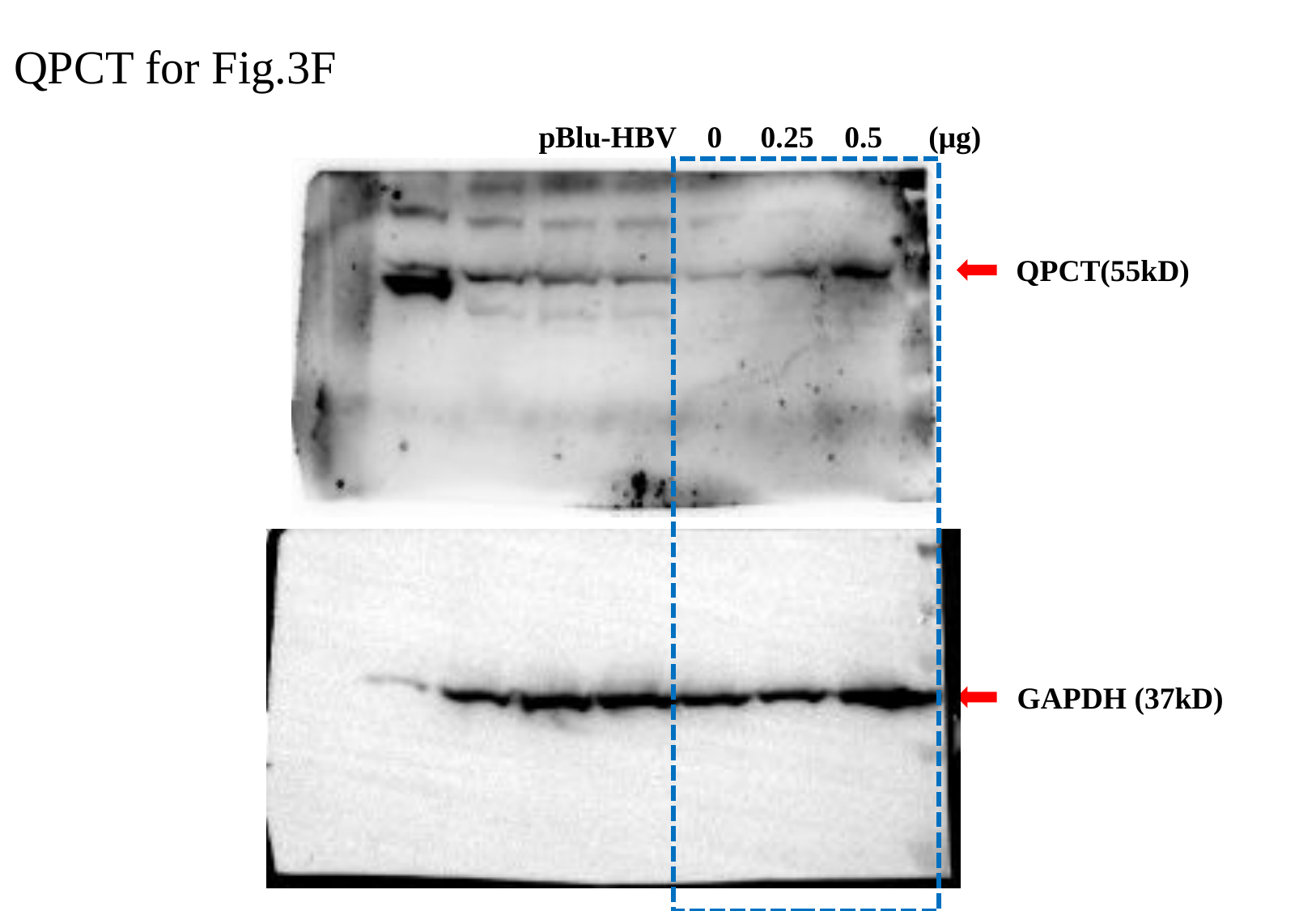

QPCT for Fig.3F
pBlu-HBV 0 0.25 0.5 (μg)
QPCT(55kD)
GAPDH (37kD)

## Slide 3
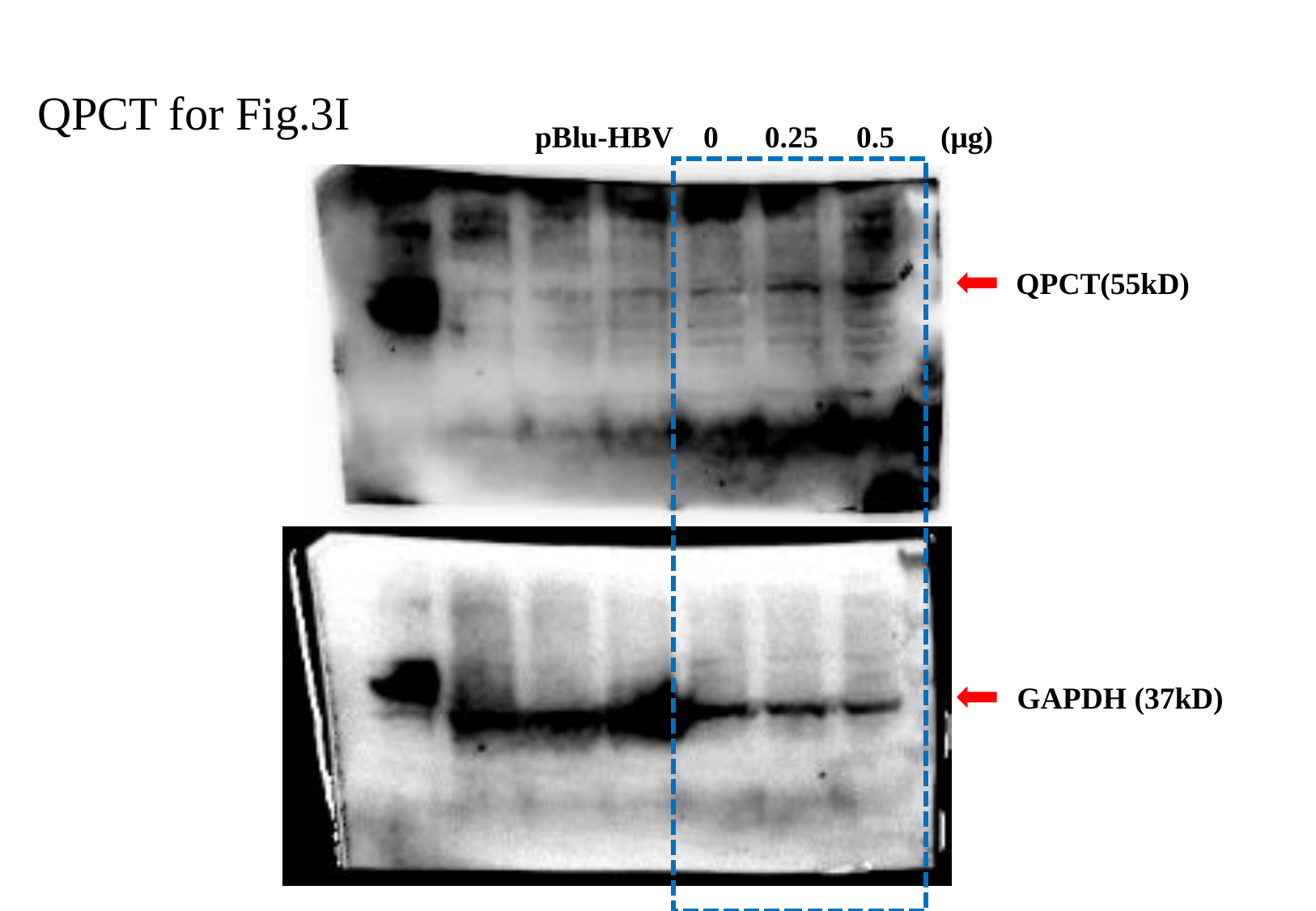

QPCT for Fig.3I
pBlu-HBV 0 0.25 0.5 (μg)
QPCT(55kD)
GAPDH (37kD)

## Slide 4
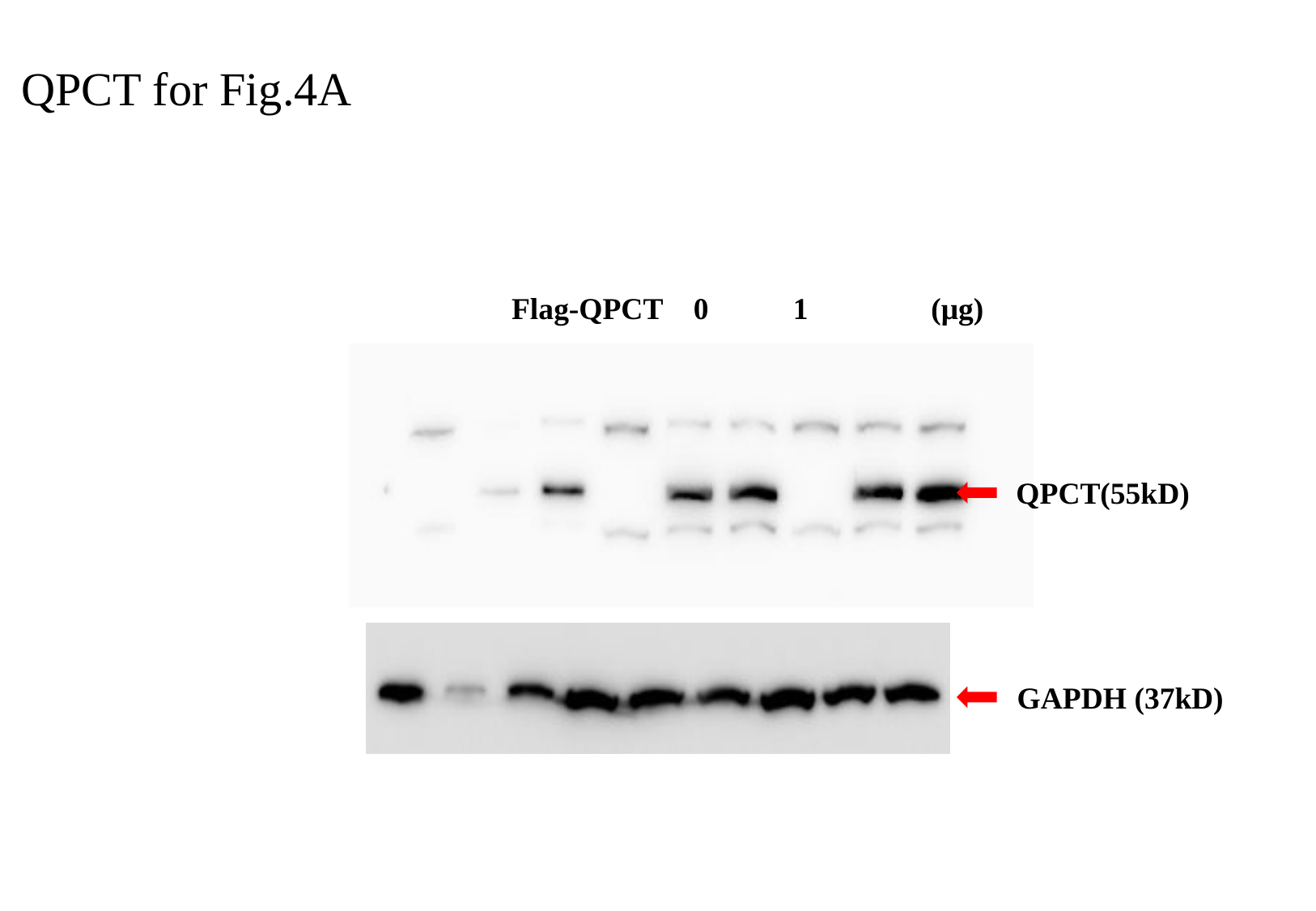

QPCT for Fig.4A
Flag-QPCT 0 1 (μg)
QPCT(55kD)
GAPDH (37kD)

## Slide 5
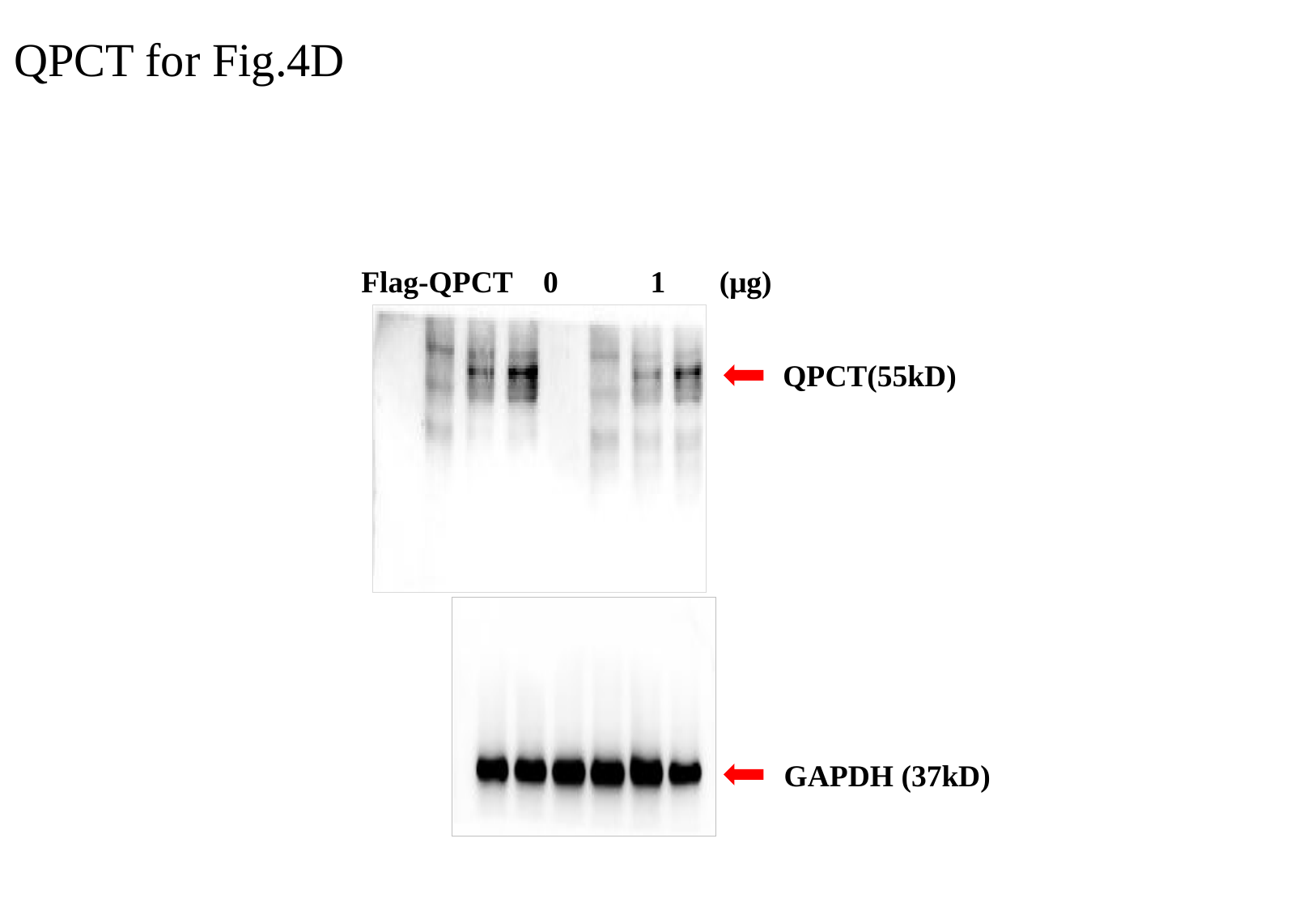

QPCT for Fig.4D
Flag-QPCT 0 1 (μg)
QPCT(55kD)
GAPDH (37kD)
